# Identification and Characterization of Novel Candidate Effector Proteins from *Magnaporthe oryzae*

**DOI:** 10.3390/jof9050574

**Published:** 2023-05-15

**Authors:** Di Liu, Zhiqin Lun, Ning Liu, Guixin Yuan, Xingbin Wang, Shanshan Li, You-Liang Peng, Xunli Lu

**Affiliations:** MOA Key Laboratory of Pest Monitoring and Green Management, China Agricultural University, Beijing 100193, China; liudi1214@163.com (D.L.);

**Keywords:** *Magnaporthe oryzae*, secreted effector, cell death, ROS, pathogenicity

## Abstract

The fungal pathogen *Magnaporthe oryzae* secretes a large number of effector proteins to facilitate infection, most of which are not functionally characterized. We selected potential candidate effector genes from the genome of *M. oryzae*, field isolate P131, and cloned 69 putative effector genes for functional screening. Utilizing a rice protoplast transient expression system, we identified that four candidate effector genes, *GAS1*, *BAS2*, *MoCEP1* and *MoCEP2* induced cell death in rice. In particular, *MoCEP2* also induced cell death in *Nicotiana benthamiana* leaves through Agrobacteria-mediated transient gene expression. We further identified that six candidate effector genes, *MoCEP3* to *MoCEP8*, suppress flg22-induced ROS burst in *N. benthamiana* leaves upon transient expression. These effector genes were highly expressed at a different stage after *M. oryzae* infection. We successfully knocked out five genes in *M. oryzae*, *MoCEP1*, *MoCEP2*, *MoCEP3*, *MoCEP5* and *MoCEP7*. The virulence tests suggested that the deletion mutants of *MoCEP2*, *MoCEP3* and *MoCEP5* showed reduced virulence on rice and barley plants. Therefore, those genes play an important role in pathogenicity.

## 1. Introduction

Plant pathogens including viruses, bacteria, oomycetes and fungi co-evolve with host plants in nature. To enable proliferation, plant pathogens secrete a large number of proteins into extracellular space or inside host cells to overcome physical barriers, subvert plant immunity, recruit nutrients and facilitate colonization. Those secreted proteins include plant cell wall degradative enzymes, virulence factors, avirulence effectors, toxins and elicitors [1,2,3,4,5]. To restrict pathogen growth, plants have evolved two layers of plant immunity. Firstly, host plasma membrane localized pattern recognition receptors (PRRs) detect extracellular-pathogen-associated molecular patterns (PAMPs) to trigger PAMP-triggered immunity (PTI); secondly, plant intracellular resistant proteins recognize avirulence effectors of pathogens to trigger effector-triggered immunity (ETI). The two plant immunity systems activate similar downstream signaling pathways with different strengths of output response [6,7].

Different from the secreted enzymes that contain notable function domains in cell wall degradation [8], most of the small secreted proteins containing enriched cysteine but with a lack of sequence similarity to known function domains, called effectors, play specific roles in pathogenicity [9,10,11]. Effectors can either interact with host-susceptible proteins to promote infection, or interfere with host-resistant components to overcome plant immunity. For example, the *Puccinia striiformis* effector Pst18363 promotes wheat rust disease by interacting with and stabilizing the negative immune factor TaNUD23 of wheat [12], while the *Magnaporthe oryzae* effector AvrPiz-t targets the rice RING-type E3 ligases APIP6 and APIP10 to suppress rice PTI [13,14]. Besides, AvrPiz-t structurally mimics rice RESISTANCE OF RICE TO DISEASES1 (ROD1), a Ca^2+^ sensor that activates a ROS-scavenging cascade, to suppress rice immunity [15]. Some effectors localize at the extracellular space surrounding host cells, such as the apoplastic proteases Sep1 and Mep1 from *Fusarium oxysporum*, which target a chitinase of tomato to destroy its function in fungal cell wall degradation [16]. Others enter into the host cells to target plant immune components, such as the *Pseudomonas syringae* HopAO1, which reduces the tyrosine phosphorylation of FLS2, a classical PRR in PTI signaling [17]. Some even enter the host cell nucleus to interfere with host transcription, for example, two nuclear effectors of *M. oryzae*, MoHTR1 and MoHTR2, function as transcription repressors to reprogram the expression of rice defense genes [18]. In turn, some apoplastic effectors trigger PTI in the host, such as XEG1, a virulence factor of *Phytophthora sojae*, which also acts as a novel PAMP to trigger innate immunity in soybean [19]. In addition, a group of cytoplasmic effectors are recognized by the plant R protein to trigger strong host cell death responses. For example, the RXLR effector AVR3a is recognized inside the cytoplasm of *Nicotiana benthamiana* and triggers a R3a-mediated hypersensitive response [20]. Overall, pathogen effector proteins often interact with host proteins, and this interaction facilitates either the pathogenicity of pathogens, or the immunity of host plants.

To understand the basis of pathogenicity, extensive studies have been carried out to identify novel effectors from different pathogens by characterizing their unique infection structures. Bacterial pathogens often use a type III secretion system (T3SS) to secrete effectors into host cells for colonization, such that the T3SS-deficient mutant *hrcC* completely lost virulence [21]. While fungal and oomycete pathogens penetrate the host cell wall and thereafter differentiate into parasitic structures such as invasive hyphae or haustoria, which are the key sites for secreting effectors into the apoplast before translocation into host cells during the infection process [22,23]. Microarrays or RNAseqs from infected leaf samples enriched with infection hyphae or haustoria have been performed to identify candidate effector proteins in several plant pathogens like *Magnaporthe oryzae*, *Blumeria graminis* and *Phytophthora phaseoli* [24,25,26,27].

The filamentous ascomycete *Magnaporthe oryzae* is the causal agent of blast disease, the most devastating disease of rice and other cereal crops, and especially the newly emerged wheat blast disease, which has become a serious threat to global wheat production [28,29,30]. Blast infection is initiated when an asexual spore adheres to the host surface, emerges from the germination tube and forms a special infection structure called appressorium to facilitate a penetration peg formation. The penetration peg then enters the host cells as a needle and differentiates to an initial bulbous infectious hypha, then develops into multiple-branched infectious hyphae within the first invaded plant cell, before expanding into neighboring cells [31,32]. Through genomic sequence analysis combined with RNAseq data of infection, more than 800 proteins are considered as potential candidate effectors [25,33,34,35,36]. Among these, around 50 effectors have been characterized, including different avirulence (Avr) effectors such as PWL1, PWL2, AVR-Pii, AVR-Pik, AVR-Pia, Avr-CO39, AVR-Pita, Avr-Pi9, AvrPiz-t and AVR-Pib [34,37,38,39,40,41,42,43]; a few virulence effectors, i.e., Slp1, MoChia1, MoNIS1, MoHTR1, MoHTR2, GAS1 and GAS2 [18,44,45,46,47]; and four biotrophy-associated secreted proteins, BAS1 to BAS4 [24]. Several effectors have been identified as cell death inducers such as MoCDIP1-13, MoNLP1, MoNLP2, MoNLP4, MoHrip1 and MoHrip2 [48,49,50,51,52]; and as cell death suppressors like SPD2, SPD4, SPD7, SPD8, SPD9 and SPD10 [53]. However, the vast majority of effectors in *M. oryzae* have not been identified and characterized.

In this study, we selected potential candidate effector genes from the genome of *M. oryzae*, field isolate P131, and cloned 69 putative effector genes. We applied a protoplast transient expression system and identified four candidate effector proteins that induce cell death in rice protoplasts, one of which also induced cell death in *N. benthamiana* leaves through *Agrobacteria*-mediated transient expression. In addition, we identified six candidate effector proteins that suppress the flg22-induced ROS burst. We further generated gene knockout mutants for five effector genes and validated the secretory function of encoding proteins. Three mutants exhibited impaired pathogenicity on host rice and barley plants. Our report here provides an efficient strategy for functional identification of fungal candidate effector proteins that are involved in plant and fungal interactions.

## 2. Materials and Methods

### 2.1. Plant and Fungal Strain Growth Conditions

The *Magnaporthe oryzae* field strain P131 was used as a wild type strain to generate mutants. All *M. oryzae* strains were cultured on oatmeal tomato agar (OTA) plates at 28 °C [54]. The colony growth and sporulation quantity assays were performed according to the previously described methods [55]. Barley (*Hordeum vulgare* cv *E9*) and *N. benthamiana* were grown in plant growth chambers at an ambient temperature of 23 °C under a 16 h light and 8 h dark cycle. Rice (*Oryza sativa* cv *Nipponbare*) was grown at 28–30 °C under a 16 h light and 8 h dark cycle.

### 2.2. Rice Protoplast Cell Death Assay

The rice protoplast cell death assay was performed as a per previously described method [56]. The open reading frame of full-length candidate effector genes was amplified from the *M. oryzae* P131 cDNA library with the primers (Table S2) and sub-cloned into the entry vector pENTR^TM^1A (A10462, Invitrogen, Waltham, MA, USA). Then, the gateway-compatible vector pIPKb002 was used for the expression of these candidate effector genes [57]. The stem and sheath of 14-day-old etiolated rice seedlings were cut into 1.5 mm strips and soaked in a protoplast isolation buffer for 3 h in the dark with gentle shaking (50 rpm/min). Then the recombinant vectors were transfected into rice protoplast, which was suspended in transfection buffer 1 to a concentration of 3.5 × 10^5^ cells/mL. A firefly luciferase (LUC) reaction with the lysate was performed using the Luciferase Assay System (E1500, Promega, Madison, GA, USA), and the LUC activity was detected using a luminometer (F200, Tecan, Männedorf, ZRH, CH).

### 2.3. Agrobacterium Tumefaciens Infiltration Assay in N. benthamiana

The recombinant plasmids were transformed into *Agrobacterium tumefaciens* strain GV3101 for the agroinfiltration of *N. benthamiana* leaves. Agrobacterium strains were cultured in a YEB liquid medium with appropriate antibiotics and re-suspended in an infiltration buffer (10 mM MgCl_2_, 10 mM 2-[N-morpholino] ethanesulfonic (pH 5.6), 200 μM acetosyrigone) to a final OD_600_ of 1.0. After incubation in the dark for 3 h, the *Agrobacterium tumefaciens* suspensions were syringe-infiltrated into fully expanded five-week-old *N. benthamiana* leaves for transient expression.

### 2.4. Measurement of Reactive Oxygen Species (ROS)

The ROS assay was performed according to a previously described method [58] with minor modification. *N. benthamiana* leaves expressing the indicated constructs were cut into 3 mm disks and incubated overnight in sterile water under dark conditions. The discs were immersed into a 96-well plate containing 100 μL reaction mixture (50 μL sterile water, 400 mΜ luminol, 20 μg/mL peroxidase and 1 μΜ flg22). Luminescence was monitored continuously for a 30 min period at 1 min intervals by a multifunctional microplate reader.

### 2.5. RNA Extraction and Gene Expression Analysis by qRT-PCR

The total RNA was isolated from 100 mg of leaves of rice, barley or *N. benthamiana* using an RNA Kit (ZP405K, ZYMO Research, Beijing, China). A quantitative real-time reverse transcription polymerase chain reaction (qRT-PCR) was performed on an ABI7500 Fast Real-Time PCR system (Applied Biosystems Inc., Foster City, CA, USA) using a 2 × HQ SYBR qPCR Mix (ZF502-2, ZYMO Research, Beijing, China). The expression of the gene was normalized to the actin of *N. benthamiana*. The experiments were conducted with three biological replicates, and one representative result was shown. The corresponding primers are listed in Table S2.

### 2.6. Yeast Secretion Assays

The secretory ability of the candidate effectors was evaluated as described previously [59]. The vector pSUC2 contains a truncated invertase gene which lacks a start codon and signal peptide sequence. The fragments of the candidate effector genes were cloned into the *Eco*RI and *Xho*I of the vector pSUC2, and the recombinant vectors were transformed into the yeast strain YTK12. These transformants were grown on synthetic dropout medium tryptophan (SD-T) to ensure expression of the pSUC2-derived plasmids and then were plated on the YPRAA to detect invertase activity. The genes *avr1b* and *mg87* were used as positive and a negative controls, respectively [60].

### 2.7. Extraction of Secreted Proteins of M. oryzae

Extraction of the secreted protein was carried out according to a previously described method [61]. In brief, fresh mycelia were grown in liquid CM for 24 h; then the liquid medium was filtrated through Miracloth (Merck Millipore, Burlington, MA, USA). The secreted proteins in the liquid medium were precipitated by adding 12.5% (*v*/*v*) trichloroacetic acid at 4 °C overnight. The pellets were harvested after centrifuging for 30 min at 12,000 rpm, and washed twice with acetone and dried. The protein samples were detected by Western blotting.

### 2.8. Gene Deletion and Pathogenicity Assays

To construct the replacement vector of candidate effector genes, 1.4~kb upstream (LB)/downstream (RB) flanking sequences of the gene were introduced into the vector pKOV21 using a one-step cloning kit (C112, Vazyme Biotech, Nanjing, China). Then the vectors were confirmed by sequencing, and subsequently were transformed into the protoplast of P131 via PEG-mediated transformation [62]. The deletion mutants were confirmed by Southern blot hybridization.

For the virulence test, four-week-old seedlings of rice and one-week-old seedlings of barley were used for pathogenicity assays. Conidia suspension of different *M. oryzae* strains were used for spray inoculation or drop inoculation, as described previously [62]. Leaves were photographed at 5 days post inoculation (dpi), and the lesion area and lesion numbers on leaves were calculated.

## 3. Results

### 3.1. Selection of Potential Candidate Effector Genes from M. oryzae

**Figure 1:**
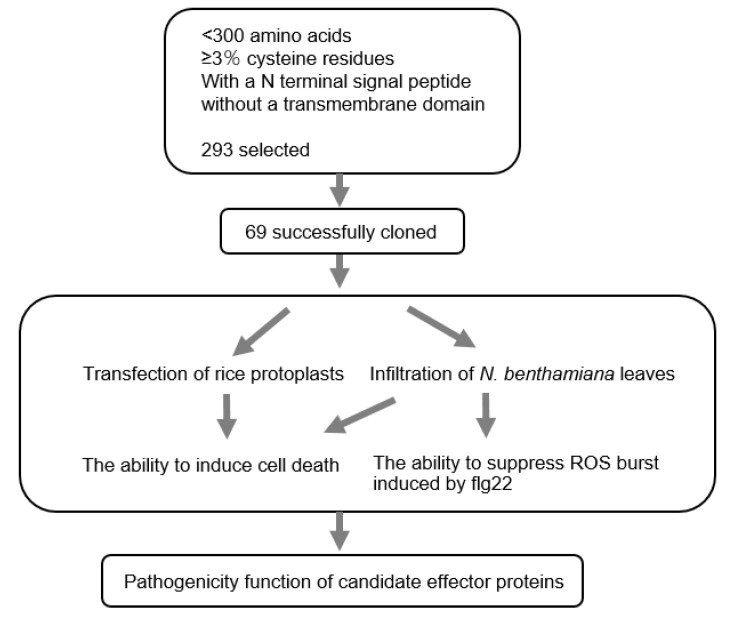
Experimental strategy for identifying potential pathogen-related effector genes from *M. oryzae*.

We have previously reported the genomic sequence from the *M. oryzae* field isolate P131. Compared with the widely used laboratory strain 70-15, the field isolate exhibited a massive expansion of gene families that are likely involved in plant–fungal interactions [63]. In this study, potential candidate effector genes were selected from the genome of the P131 isolate, and were tested in their ability to trigger cell death responses in rice protoplast, or their ability to suppress flg22-induced ROS after transient expression in *N. benthamiana* leaves. To select the potential candidate effector genes, the following criteria were used: the genes should encode proteins containing fewer than 300 amino acids with enriched cysteine residues (≥3%) and carry an N terminal signal peptide, but without a transmembrane domain [64,65]. Based on those criteria, 293 genes were selected and 69 of them were successfully cloned from cDNA generated from the P131 isolate (Table S1). The CDS of those 69 genes were initially inserted into a pENTER^TM^1A entry vector, and then into the expression vector pIPKb002 with a maize ubiquitin promoter (pUbi) for further functional tests [57]. In the following up tests, transient transformation of those genes in rice protoplasts and *N. benthamiana* leaves was performed to determine whether those genes could induce plant cell death responses. We also investigated the ability of these candidate effectors to suppress the flg22-induced ROS responses in *N. benthamiana*. Eventually, the candidate effector genes were tested for their pathogenicity function (Figure 1).

### 3.2. Identification of Four Candidate Effector Proteins That Induce Cell Death in Rice Protoplasts

**Figure 2:**
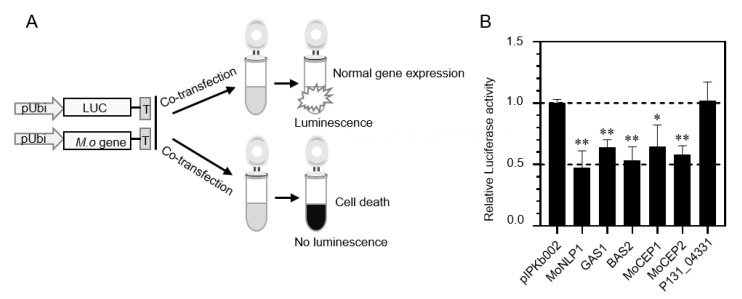
Identification of the candidate effector genes that induce cell death in rice protoplasts. (**A**) Experimental strategy for the detection of rice protoplast cell death by luciferase activity. The plasmid containing the firefly luciferase gene (*LUC*) and the plasmid containing candidate effector genes were co-transfected into rice protoplast cells. If the expressed candidate effector genes could not induce cell death, luciferase activity was strong (top). If the expressed candidate effector proteins could induce cell death, luciferase activity was weakened (bottom). (**B**) Luciferase activity of rice protoplasts was measured after transfection of the different candidate effector genes. pIPKb002 and MoNLP1 were used as an empty vector and a positive control, respectively. (Student’s *t* test, * *p* < 0.05, ** *p* < 0.01).

During plant–pathogen interactions, many pathogen effectors induce host cell death responses. To identify potential candidate effectors of *M. oryzae* that induce host cell death, we transiently expressed the full-length effector genes in rice protoplasts (Figure 2A). In this assay, the plasmids of the pUbi driving LUC reporter gene were mixed with plasmids of the individual candidate effector genes to transform rice protoplasts, and the luciferase activity was quantified for the level of cell viability [56]. The transient expression assay revealed that 4 out of 69 potential effector genes led to a significant reduction of luciferase activity when expressed in the rice protoplasts (Figure 2B). The cell death responses were similar to that caused by the positive control MoNLP1, which is a well-known cell death inducer [50]. However, rice protoplasts exhibited normal luciferase activity when other tested genes were transiently expressed, such as P131_04331(MGG_00245) (Figure 2B). The four genes that induced cell death in the rice protoplast are P131_00405(MGG_12337), P131_09587(MGG_09693), P131_00603(MGG_08086) and P131_04657(MGG_05232). Of these, P131_00405 encodes GAS1, a function effector protein highly enriched in appressoria, and P131_09587 encodes BAS2, a secreted protein specifically localized to BICs [24,47]. The other two genes, P131_00603 and P131_04657, were thus named as *MoCEP1* and *MoCEP2* (*M. oryzae* **c**andidate **e**ffector **p**roteins) in this study. There is no single nucleotide polymorphism (SNP) in the CDS sequences of these four genes among P131, 70-15 and other field isolates.

### 3.3. One Candidate Effector Protein Induces Cell Death in N. benthamiana

**Figure 3:**
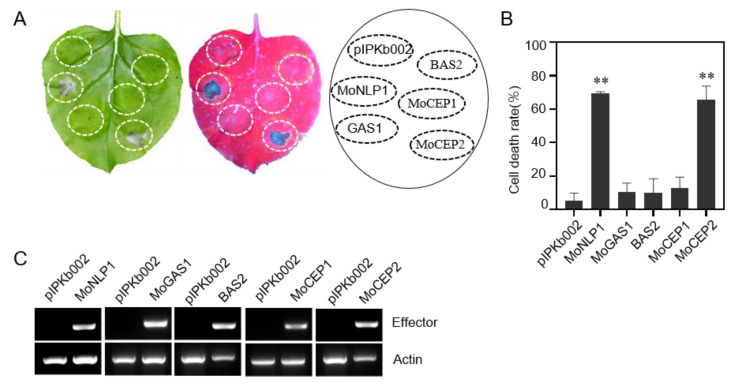
Identification of one candidate effector gene that elicits cell death in *N. benthamiana*. (**A**) *N. benthamiana* leaves infiltrated with Agrobacterium strains carrying different candidate effector genes were photographed under visible and ultraviolet lights at 5 days post infiltration (dpi). pIPKb002, an empty vector, was used as a negative control, and MoNLP1 was used as a positive control. (**B**) Statistical analysis of the cell death rate of *N. benthamiana* leaves. The experiment was repeated at least three times, n = 30 (Student’s *t* test, ** *p* < 0.01). (**C**) The expression of candidate effector genes was detected by semi-quantitative RT-PCR in *N. benthamiana*. Total RNA was extracted from *N. benthamiana* leaves at 36 h post *Agrobacteria* injection. Actin was used as a positive control.

Previous reports on screening potential *M. oryzae* effectors have been based on the observation of non-host cell death in *N. benthamiana* [48,49,66]. We also tested whether those potential candidate effectors can trigger non-host cell death in *N. benthamiana* via an *Agrobacterium*-mediated transient expression approach. Among the 69 potential candidate effector genes, only *MoCEP2* induced cell death responses in the *N. benthamiana* leaves. Surprisingly, the genes *GAS1*, *BAS2* and *MoCEP1* that induce cell death in rice protoplast, could not induce cell death responses in *N. benthamiana* leaves (Figure 3A,B). RT-PCR analysis verified that those genes were expressed in *N. benthamiana* (Figure 3C). Together, these results imply that only *MoCEP2* induces both host cell death and non-host cell death responses.

### 3.4. Identification of Six Candidate Effector Proteins That Suppress flg22-Triggered ROS Burst in N. benthamiana

**Figure 4:**
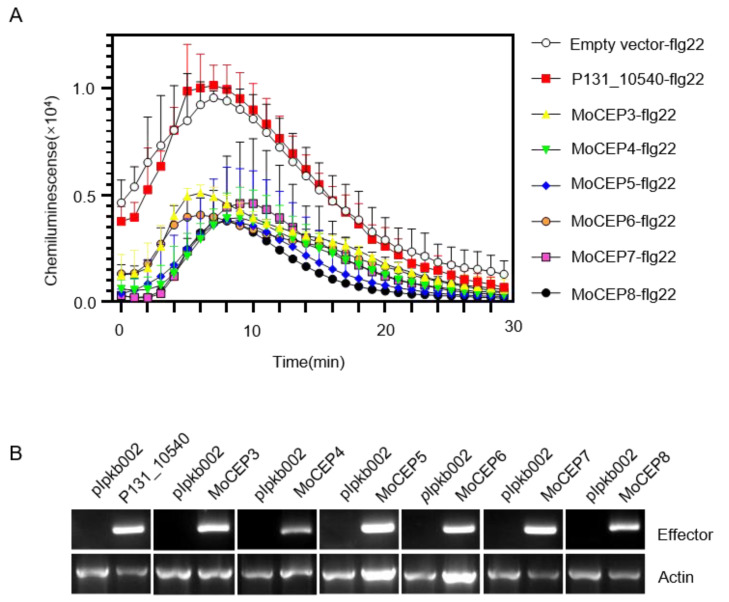
Identification of the candidate effector genes that suppress a flg22-triggered ROS burst in *N. benthamiana* after transient expression. (**A**) The candidate effector genes were transiently expressed in *N. benthamiana* leaves, and then the reactive oxygen species (ROS) generated in *N. benthamiana* leaf discs treated with 100 nm flg22 were measured. Error bars denote standard deviations from three replicates. The empty vector and the effector gene *P131_10540* served as negative controls. (**B**) The expression of candidate effector genes was detected by semi-quantitative RT-PCR in *N. benthamiana*. Total RNA was extracted from *N. benthamiana* leaves at 36 h post *Agrobacteria* injection. Actin was used as a positive control.

For successful colonization, pathogens secrete virulence effectors to suppress host defense responses, such as PAMP-triggered ROS production [67,68,69]. In this study, we evaluated whether a flg22-induced ROS burst was suppressed by the expression of the potential candidate effector genes in *N. benthamiana* leaves. When expressed in *N. benthamiana*, 6 out of the 69 genes inhibited the flg22-induced ROS burst, whereas the other tested genes such as P131_10540(MGG_08428) could not (Figure 4A). In the *N. benthamiana* leaves expressing all those genes, water treatment could not induce ROS response (Figure S1). These six genes are P131_02098(MGG_03671), P131_07667(MGG_09019), P131_08608(MGG_15401), P131_10190(MGG_09842), P131_04965(MGG_04944) and P131_11327(MGG_02715), named as *MoCEP3* to *MoCEP8*, respectively. Additionally, BLAST searches revealed that one SNP was present in the CDS sequences of *MoCEP5* and *MoCEP7* among the laboratory strain 70-15 and the field isolates P131, MO004 and MO102 (Figure S2), while no SNP was present in the other four *CEP* genes. RT-PCR analysis verified that those genes were expressed in *N. benthamiana* (Figure 4B). These six CEPs may play important roles in suppressing host immunity against pathogens.

### 3.5. Nine Candidate Effector Protein Genes Are Highly Expressed during M. oryzae Infection

**Figure 5:**
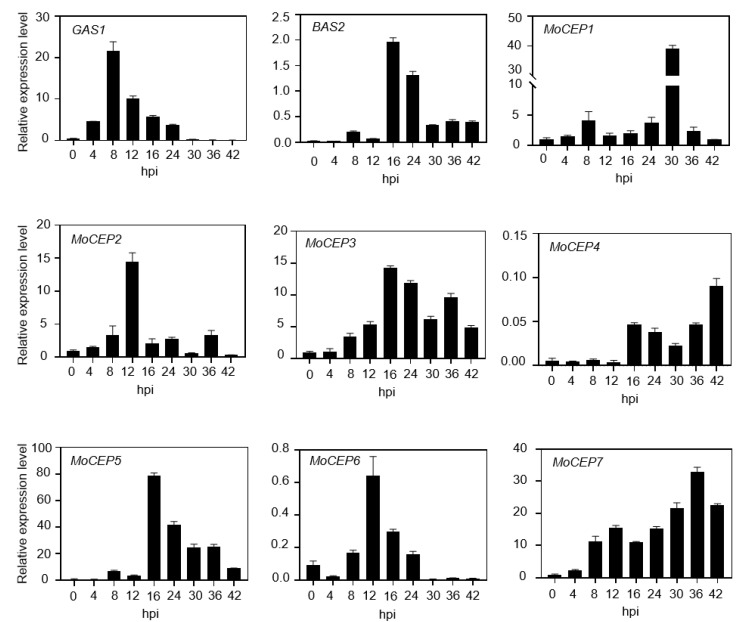
The relative expression level of nine candidate effector genes at different blast infection stages. Barley leaves were spray-inoculated with P131 conidial suspension (5 × 10^5^ conidia/mL) and the total RNA was extracted from barley leaves at indicated hours post inoculation (hpi). The expression of nine candidate effector genes relative to actin was calculated by RT-qPCR.

In total, we have identified ten candidate effector protein genes, including cell death inducers *GAS1*, *BAS2*, *MoCEP1* and *MoCEP2*, and ROS suppressors *MoCEP3* to *MoCEP8*. To evaluate the expression pattern of the ten genes in infected leaves, qRT-PCR was carried out using the RNA extracted from barley leaves inoculated with P131 isolate at different time points. The signal of *MoCEP8* was not observed, possibly because of a low proportion of fungal mass in the infected leaves. The expression of the other nine genes was at a relative low level at the initial time of inoculation (0 h), but strongly increased during the fungal infection. Specifically, *GAS1* was highly expressed at 8 hpi, *MoCEP2* and *MoCEP6* exhibited the highest expression at 12 hpi, while *BAS2*, *MoCEP3* and *MoCEP5* did so at 16 hpi. By contrast, *MoCEP1*, *MoCEP4* and *MoCEP7* showed a significant increase at the later infection stage (30–42 hpi) (Figure 5). Overall, nine out of ten candidate effector genes were expressed specifically in the initial period of *M. oryzae* infection, suggesting that these candidate effector proteins function in pathogenicity.

### 3.6. Validation of the Secretory Function of Five Candidate Effector Proteins

Since the function of *GAS1* and *BAS2* has been reported [24,47], we mainly characterized the function of the other candidate effector genes through generating gene deletion mutants in *M. oryzae* via targeted gene replacement. Finally, we successfully generated gene deletion mutants for *MoCEP1*, *MoCEP2*, *MoCEP3*, *MoCEP5* and *MoCEP7*. The mutants for these five genes were confirmed by Southern blot analysis (Figure S3). Bioassays showed that the mutants of those candidate effector genes are similar to the wild-type strain P131 in mycelial growth and conidiation (Figure S4).

**Figure 6:**
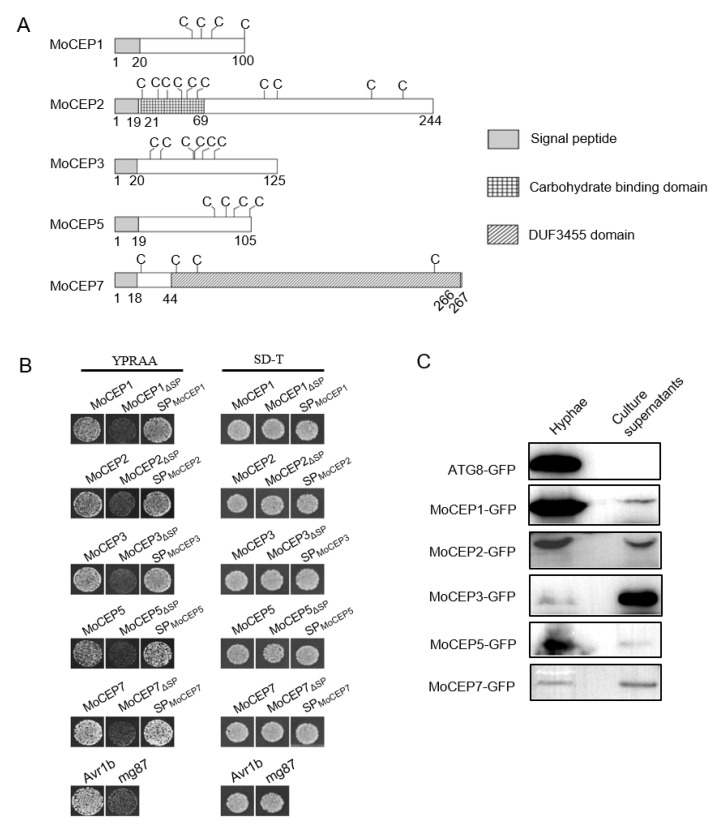
The secretory function of five candidate effector proteins. (**A**) Deduced protein domains of the five candidate effector proteins. The letter C indicates the cysteine. (**B**) Detection of the secretory function of candidate effector protein signal peptides through yeast secretion assay. Avr1b and mg87 were used as a positive and negative control, respectively. (**C**) Immunoblotting analysis of the five candidate effector proteins from the growth hyphae and liquid media using anti-GFP antibody. Atg8-GFP was used as a control protein that was expressed only in the growth hyphae but not in liquid media.

We analyzed the domain structure of the five candidate effector proteins using SMART (http://smart.embl-heidelberg.de/, accessed on 1 October 2022) and found that they all contain a predicted N-terminal signal peptide (SP). MoCEP2 contains a carbohydrate binding domain (CBD), which shares partial similarity to the CBD of *Schizosaccharomyces pombe* endo-1,3-b-glucanase Eng1 [70], MoCEP7 contains a functionally uncharacterized DUF3455 domain, while the MoCEP1, MoCEP3 and MoCEP5 proteins do not have predicted domains (Figure 6A).

To experimentally verify the predicted signal peptides, a yeast signal trap assay was utilized. The predicted SP nucleotide sequence, the full-length effector coding sequence and the truncated protein without the predicted SP sequence of each gene were fused to the truncated *SUC2* gene lacking its original signal peptide coding sequence. The fusion constructs were transformed into an invertase-deficient yeast strain YTK12, thus only the yeast cells expressing functional signal peptides could hydrolyze raffinose and grow on YPRAA media such as the positive control Avr1b [71,72]. Consistent with the prediction, the yeast transformed with the full-length effector proteins or the predicted signal peptides of each protein did grow on YPRAA media, but the yeast transformed with the truncated proteins without the predicted SP sequence did not (Figure 6B), suggesting that the signal peptides were functional. To further determine whether these effectors are secretory proteins, individual constructs containing the *MoEGF1* promoter and the CDS of each gene with a C-terminal GFP tag were generated and transformed into wild-type strain P131 [73]. The fusion gene transformants were cultured in liquid CM, and the presence of effector proteins were detected both in the mycelium and medium liquid for all five candidate effector proteins in the immunoblots using an anti-GFP antibody (Figure 6C). Collectively, we demonstrated that MoCEP1, MoCEP2, MoCEP3, MoCEP5 and MoCEP7 are bona fide secreted proteins.

### 3.7. Three Candidate Effector Genes Are Virulence Factors in M. oryzae

**Figure 7:**
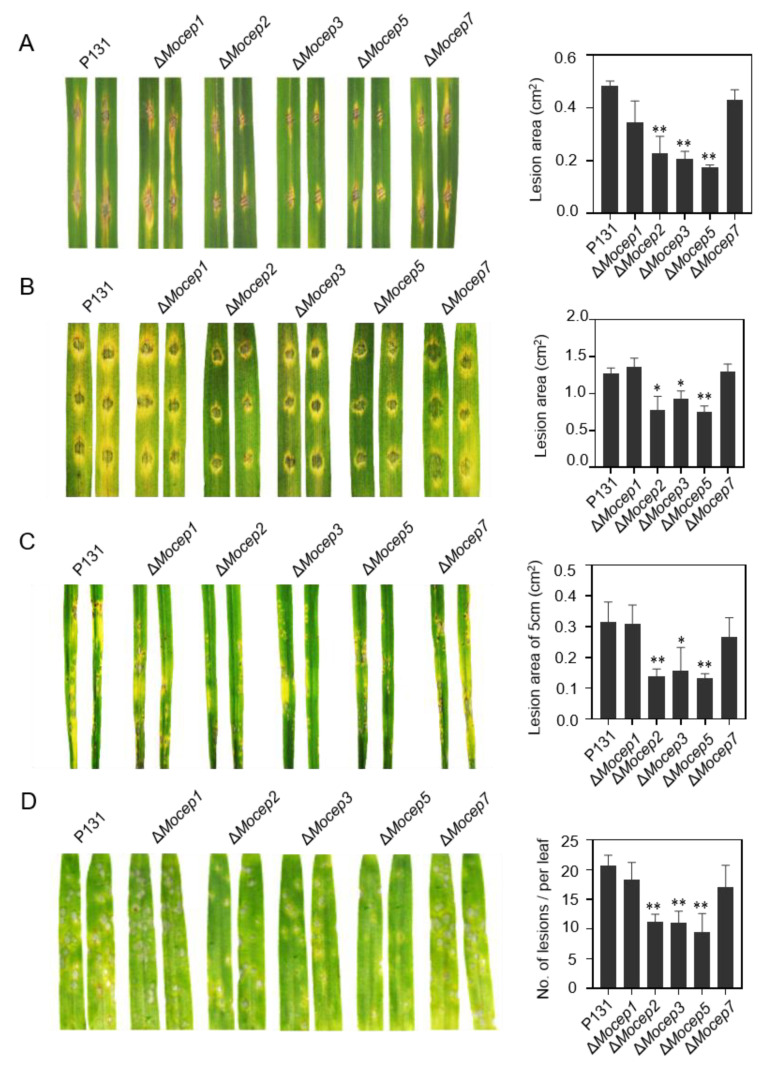
Three effector genes are important for fungal virulence. (**A**) Scratched rice leaves drop-inoculated with 10uL conidial suspension (1 × 10^5^ condia/mL) of the indicated strains were photographed at 5 dpi, and the lesion area was calculated, n = 18 (Student’s *t* test, ** *p* < 0.01). (**B**) Barley leaves drop-inoculated with 10uL conidial suspension (5 × 10^4^ condia/mL) of the indicated strains were photographed at 5 dpi, and the lesion area was calculated, n = 18 (Student’s *t* test, * *p* < 0.05, ** *p* < 0.01). (**C**) Rice leaves sprayed with 5mL conidia suspension (1 × 10^5^ condia/mL) of the indicated strains were photographed at 5 dpi, and the lesion area was calculated, n = 6 (Student’s *t* test, * *p* < 0.05, ** *p* < 0.01). (**D**) Barley leaves sprayed with 2 mL conidia suspension (2 × 10^4^ condia/mL) of the indicated strains were photographed at 5 dpi, and the number of lesions was calculated, n = 6 (Student’s *t* test, ** *p* < 0.01).

Previous studies have reported that *GAS1* is a virulence factor in *M. oryzae* [47], but the disruption of *BAS2* does not show phenotypes in pathogenicity [24]. To characterize the function of the five candidate effector genes in pathogenicity, a conidial suspension of P131 and each effector gene mutant were inoculated onto rice and barley leaves via drop-inoculation or spray-inoculation methods. The strains ∆*Mocep2*, ∆*Mocep3* and ∆*Mocep5* caused a significantly reduced lesion area compared with P131 on both rice and barley leaves, while strains ∆*Mocep1* and ∆*Mocep7* exhibited normal virulence as P131 (Figure 7). These data demonstrate that *MoCEP2*, *MoCEP3* and *MoCEP5* play important roles in fungal pathogenicity.

## 4. Discussion

In this study, we cloned 69 *M. oryzae* genes encoding potential candidate effector proteins for functional screening. Among those 69 candidate effector genes, transient expression of four genes, *GAS1*, *BAS2*, *MoCEP1* and *MoCEP2*, induced cell death responses in rice protoplast, and *MoCEP2* also led to a necrosis phenotype in *N. benthamiana* when expressed transiently. Furthermore, the transient expression of six genes, *MoCEP3* to *MoCEP8*, suppressed the flg22-induced ROS burst in *N. benthamiana*. Finally, we generated gene knockout mutants of five candidate effector genes in *M. oryzae*, and revealed that *MoCEP2*, *MoCEP3* and *MoCEP5* function as key factors in fungal pathogenicity. Together, the functional screening approaches help us to identify novel candidate effector genes from *M. oryzae*.

Cell death responses are commonly observed when pathogens grown in resistant host or non-host plants, and are closely associated with the induction of plant immunity [74]. Many cell-death-inducing proteins from various pathogens have been characterized based on heterogeneous expression, especially using the *Agrobacterium*-mediated transient expression in *N. benthamiana* [75,76]. Due to possible differences between the immune systems of monocot and dicot plants, the effector proteins of a certain pathogen that cause cell death in *N. benthamiana* may not be essential to its infection of a host. For example, for the cell-death-inducer genes *VdSCP27*, *VdSCP113* or *VdSCP126* in *Verticillium dahlia*, the knockout of individual genes did not change the fungal virulence on susceptible cotton [11]. Therefore, it is necessary to directly test the candidate effector proteins of a pathogen in its host cells. In this project, we carried out the screening of cell death phenotype in host protoplast cells [56]. Four cell-death-inducing proteins were identified (Figure 2B), among which *GAS1* was reported to be expressed specifically in appressoria and is essential for plant infection [47] and *BAS2* was specifically expressed in invasive hyphae in a previous report [24]. Apart from the two previously reported effector genes, *MoCEP1* and *MoCEP2* are novel candidate effector genes that induce cell death in rice protoplasts. *MoCEP2* also induced cell death responses in *N. benthamiana* leaves (Figure 3A), and the knockout mutant ∆*Mocep2* exhibited reduced virulence on rice and barley plants (Figure 7). Both *GAS1* and *MoCEP2* are key players in fungal pathogenicity, indicating that our strategy of testing for a cell death phenotype in rice protoplast is efficient for identifying functional effector proteins. However, the knockout mutant ∆*Mocep1* does not exhibit a notable phenotype in virulence (Figure 7), similar to the MoNLPs family genes that induce cell death on *N. benthamiana* leaves. Also, among the 13 cell-death-inducing proteins, MoCDIP1 to MoCDIP13, that induced cell death in rice protoplasts or on *N. benthamiana* leaves [48,49], only MoCDIP4 functions as a virulence factor [77]. Taken together, most candidate effector proteins contribute minor roles to fungal pathogenicity [50]. Notably, in vitro purified MoNLPs, and CDIP6/7 proteins activate plant immunity against rice blast fungus. Therefore, further investigation is needed to test whether BAS2 and MoCEP1 proteins also have a similar function.

Reactive oxygen species (ROS) act as key signals or toxic molecules with strong oxidant power during plant–pathogen interactions [78,79]. Plants react rapidly and produce a transient ROS burst in PTI, whereas the ROS production in ETI is much more persistent [80]. In order to establish successful infection, pathogens secrete a variety of effectors to impair host immune responses including ROS production. Examples are *Colletotrichum* CoNIS1, ChNIS1 and *M.oryzae* MoNIS1 [46], *Fusarium oxysporum* effectors Six1, Foa1, Foa2 and Foa2 [81], *Acidovorax citrulli* effector AopV [82] and *Pseudomonas syringae* type III effectors HopAI1 and HopF2 [83,84]. Here, we screened the suppressors of the flg22-induced ROS responses in *N. benthamiana* transient expressed potential candidate effector genes. Six candidate effector genes were identified expressing ROS suppressors (Figure 4A), and the knockout mutants *∆Mocep3*, *∆Mocep5* and *∆Mocep7* were generated in the P131 background for the pathogenicity test. Subsequently, we assessed that *MoCEP3* and *MoCEP5* play key roles in virulence (Figure 7). A few pathogen effectors directly target PTI signaling components to prevent host ROS production. For example, the *Xanthomonas campestris* type III effector AvrAC and the *M. oryzae* effector MoNIS1 target different receptor-like cytoplasmic kinases to inhibit PTI signaling [46,85], whereas the *Phytophthora sojae* effector CRN78 phosphorylates and leads to the degradation of aquaporin NbPIP2;2 to suppress ROS accumulation in *N. benthamiana* [69]. The candidate effector proteins MoCEP3 and MoCEP5 might also target key players in PTI signaling, which needs further investigation.

Ten modules of gene expression patterns were characterized corresponding to different infection life cycles of *M. oryzae* [25]. In our study, nine candidate effector genes have distinct gene expression patterns belonging to four modules (Figure 5), suggesting that these genes may have different functions or be regulated with distinct mechanisms, such as the genome regulation mediated by histone modification, or transcriptional regulation by Rgs1 [86,87]. Consistent with the previous observation, we confirmed that *GAS1* showed peak expression at 8 hpi, which was associated with an appressorium-mediated infection as module 2 genes. *BAS2* was highly expressed at 16 hpi, and functioned at infection hyphae elongation as module 3 genes. In addition, the expression of *MoCEP2*, *MoCEP3*, *MoCEP5* and *MoCEP6* showed peaks at 12–16 hpi, indicating that they belong to module 4 and might function at the initial biotrophic stage. In contrast, the expression of *MoCEP1*, *MoCEP4* and *MoCEP7* exhibit peaks at 30–42 hpi, suggesting that they may function at the transition stage to necrotrophic colonization. However, the knockout mutants ∆*Mocep1* or ∆*Mocep7* did not show any phenotype related to fungal virulence. It is possible that the detection system we used was unable to accurately measure small changes in pathogenicity. A relative fitness assay has been applied to test the function of *Mep1* in virulence [25], and the same approach could be used for our candidate effector genes. In addition, generating multiple gene knockout mutants has addressed the functional complexity of fungal virulence factors [88], which would be an effective approach to test the function of *M. oryzae* virulence genes in the future.

For three candidate effector protein genes *MoCEP4*, *MoCEP6* and *MoCEP8*, we could not obtain gene knockout mutants via a homogenous recombination approach, and a similar situation has also applied to effector gene BAS4 [24], possibly because of retrotransposon elements flanking the gene coding region. Since CRISPR-Cas9-mediated BAS4 editing resulted in multiple BAS4 deletion mutants of *M. oryzae*, CRISPR techniques could be used as an alternative approach to generate mutants for those candidate genes in the future [89].

## 5. Conclusions

To subvert plant immunity, *M. oryzae* secretes a large number of effectors to manipulate host immune responses; however, the vast majority of effectors have not been identified and characterized. Our study cloned 69 *M. oryzae* putative effector genes for functional screening. Four cell death inducers, *GAS1*, *BAS2*, *MoCEP1* and *MoCEP2*, were identified by utilizing the rice protoplast transient expression system, and *MoCEP2* also induced cell death in *N. benthamiana* leaves through *Agrobacteria*-mediated transient expression. In addition, we identified that the transient expression of six candidate effector genes, *MoCEP3* to *MoCEP8*, suppressed the flg22-induced ROS burst. Pathogenicity tests in gene knockout mutants revealed that *MoCEP2*, *MoCEP3* and *MoCEP5* are virulence factors during fungal infection. Our study found valuable candidate effector proteins for further investigation on the molecular mechanisms underlying the rice–blast fungus interaction.

## Data Availability

The data presented in this study are included in the article; further inquiries can be directed to the corresponding author.

## Supplementary Materials

The following supporting information can be downloaded at: https://www.mdpi.com/article/10.3390/jof9050574/s1, Figure S1: Water treatment could not induce ROS burst in *N. benthamiana* leaves. Figure S2: (A,B) The alignment of CDS of *MoCEP5* (A) and *MoCEP7* (B) from the laboratory strain 70-15 and the field isolates P131, MO004 and MO102. Figure S3: The knockout of candidate effector genes. Figure S4: The five candidate effectors are dispensable for vegetative growth and sporulation of *M. oryzae*. Table S1: List of 69 cloned putative effector genes from *M. oryzae* field isolate P131. Table S2: Primers used in this study.
