# Supplementary material for: Identification and Characterization of Novel Candidate Effector Proteins from Magnaporthe oryzae
Source: J Fungi (Basel). 2023 May 15;9(5):574. doi: 10.3390/jof9050574 (PMC10219149; doi:10.3390/jof9050574)
Supplement: Supplementary file 1 [file jof-09-00574-s001.zip › Supplemental materials legends .pdf]

Figure S1. Water treatment could not induce ROS burst in *N. benthamiana* leaves. The candidate effector genes were transiently expressed in *N. benthamiana* leaves, and then the reactive oxygen species (ROS) generated in *N. benthamiana* leaf discs treated with H_2O were measured. Error bars denote standard deviations from three replicates.

Figure S2. (A and B) Nucleotide sequence alignment of candidate effector genes *MoCEP5* (A) and *MoCEP7* (B) between the laboratory strain 70-15 and the field isolates P131, DG23 and QSP9-1-3. The substitutions of nucleotide in the four distinct strains are highlight in red. I, Isoleucine; V, Valine; F, Phenylalanine;

Figure S3. The knockout mutants of candidate effector genes.
(A) Schematic diagram of the gene deletion strategy for five candidate effector genes. HPT, hygromycin phosphotransferase gene. X, *Xho*I; P, *Pst*I; B, *Bam*HI. (B) DNA gel blot analysis of the knockout mutants using the digoxin-marked hybridization probes.

Figure S4. The five candidate effector genes are dispensable for the vegetative growth and sporulation of *M. oryzae*.
(A) The knockout mutants of five candidate effector genes grow normally on oatmeal-tomato agar plates (OTA). Five-day-old oatmeal–tomato agar cultures of wild-type strain P131 and mutant strains were photographed. (B) The colony growth diameters were measured. The experiment was repeated three times. (C) Statistical analysis of sporulation from strains P131 and deletion mutants of five effector genes on OTA plates. Error bars denote standard deviations from three replicates.
