# Supplementary material for: Identification and Characterization of Novel Candidate Effector Proteins from Magnaporthe oryzae
Source: J Fungi (Basel). 2023 May 15;9(5):574. doi: 10.3390/jof9050574 (PMC10219149; doi:10.3390/jof9050574)
Supplement: Supplementary file 1 [file jof-09-00574-s001.zip › Table S1.pdf]

Table S1 List of 69 cloned putative effector genes from *M.oryzae* isolate P131.

No.	Gene ID	Amino Acids	Description	Comments
1	P131_04331	183	Chitin binding protein	
2	P131_08798	229	Chitin binding protein	
3	P131_00405	251	MAS3 protein	GAS1
4	P131_10209	283	Beta-glucanase	
5	P131_02081	153	Avr-Pi54 protein	
6	P131_09535	153	Extracellular serine-rich protein	
7	P131_06853	290	MAS3 protein	
8	P131_03335	117	Hypothetical protein	
9	P131_09450	132	Hypothetical protein	
10	P131_09587	102	Biotrophy-associated secreted protein 2	BAS2
11	P131_06247	86	Hypothetical protein	
12	P131_02098	125	Hypothetical protein	Identified as MoCEP3
13	P131_11979	108	AvrPiz-t protein	
14	P131_06308	140	Hypothetical protein	
15	P131_05316	286	Hypothetical protein	
16	P131_07132	114	Hypothetical protein	
17	P131_08408	118	Hypothetical protein	
18	P131_07667	193	Secretory phospholipase A2	Identified as MoCEP4
19	P131_08852	141	Hypothetical protein	
20	P131_04799	130	Hypothetical protein	
21	P131_10540	111	Hypothetical protein	
22	P131_02490	134	Hypothetical protein	
23	P131_03396	220	PCI domain-containing protein	
24	P131_08608	105	Hypothetical protein	Identified as MoCEP5
25	P131_04934	272	Carbonate dehydratase	
26	P131_10366	187	Carbohydrate-binding WSC domain-containing protein	
27	P131_00603	100	Hypothetical protein	Identified as MoCEP1
28	P131_12512	89	Hypothetical protein	
29	P131_05618	160	Hypothetical protein	
30	P131_09418	106	Hypothetical protein	
31	P131_03746	245	Hypothetical protein	
32	P131_08988	197	Biotrophy-associated secreted protein 2	
33	P131_01991	198	PCI domain-containing protein	
34	P131_05726	270	Thaumatin, pathogenesis-related protein	
35	P131_04657	244	Carbohydrate-binding module family 52 protein	Identified as MoCEP2

Table S1 List of 69 cloned effector genes from *M.oryzae* isolate P131.

No.	Gene ID	Amino Acids	Description	Comments
36	P131_10498	219	GPI anchored cell wall protein	
37	P131_05053	152	Hypothetical protein	
38	P131_11027	123	Hypothetical protein	
39	P131_06526	278	Hypothetical protein	
40	P131_10190	170	Hypothetical protein	Identified as MoCEP6
41	P131_04616	216	Extensin isoform X1	
42	P131_05913	274	Amidohydrolase 3	
43	P131_10596	274	(R)-specific carbonyl reductase	
44	P131_01240	177	Secretory phospholipase a2	
45	P131_10222	275	CAS1 domain-containing protein 1	
46	P131_02819	161	Bys1 family protein	
47	P131_08949	199	Hypothetical protein	
48	P131_01166	276	Cyclohexadienyl dehydratase	
49	P131_01375	156	Hypothetical protein	
50	P131_04965	267	Putative malate dehydrogenase protein	Identified as MoCEP7
51	P131_01161	220	Hypothetical protein	
52	P131_04833	240	Hypothetical protein	
53	P131_10254	130	Hypothetical protein	
54	P131_08851	212	Hypothetical protein	
55	P131_02351	245	GPI anchored protein	
56	P131_01889	246	Hypothetical protein	
57	P131_08602	65	Hypothetical protein	
58	P131_04259	295	Putative aspergillopepsin protein	
59	P131_08153	224	Endoglucanase II	
60	P131_02766	256	Concanavalin a-like lectin/glucanase domain-containing protein	
61	P131_02892	218	Hypothetical protein	
62	P131_02630	87	Hypothetical protein	
63	P131_10151	181	Hypothetical protein	
64	P131_11327	134	Putative neurofilament medium polypeptide protein	Identified as MoCEP8
65	P131_03327	184	Hypothetical protein	
66	P131_09986	130	Hypothetical protein	
67	P131_05921	152	Putative bys1 domain protein	
68	P131_07649	290	Aspergillopepsin-2	
69	P131_05538	83	Hypothetical protein	
