# Supplementary material for: Identification and Characterization of Novel Candidate Effector Proteins from Magnaporthe oryzae
Source: J Fungi (Basel). 2023 May 15;9(5):574. doi: 10.3390/jof9050574 (PMC10219149; doi:10.3390/jof9050574)
Supplement: Supplementary file 1 [file jof-09-00574-s001.zip › Table S2.pdf]

Table S2 Primers used in this study

No.	Primers	Sequence:5'-3'	Comments
1	04331-F	ccaattcagtcgactggatccATGTTCGCCAAGCTCGCCAT	F primer to amplify P131_04331
2	04331-R	ctcgagtgcggccgcgaattcTTATCCCTGGACAAAGTCA	R primer to amplify P131_04331
3	08798-F	ccaattcagtcgactggatccATGCAGTACTCCCTGCTCTC	F primer to amplify P131_08798
4	08798-R	ctcgagtgcggccgcgaattcTTAGTTGCACGACGCGCTGG	R primer to amplify P131_08798
5	00405-F	ccaattcagtcgactggatccATGTCTCTCAAGAGCTTGAT	F primer to amplify P131_00405
6	00405-R	ctcgagtgcggccgcgaattcTTAAGCGATCTCGCCGTCGC	R primer to amplify P131_00405
7	10209-F	ccaattcagtcgactggatccATGCGTGTTCTCTCCCTCGG	F primer to amplify P131_10209
8	10209-R	ctcgagtgcggccgcgaattcTTAGATTCCAACGTAGTGAG	R primer to amplify P131_10209
9	02081-F	ccaattcagtcgactggatccATGCAGTTCACCGCCACCAT	F primer to amplify P131_02081
10	02081-R	ctcgagtgcggccgcgaattcCTAGCAGCCATAGGTGAGGA	R primer to amplify P131_02081
11	09535-F	ccaattcagtcgactggatccATGCAGTTCAAGGCCGTCCT	F primer to amplify P131_09535
12	09535-R	ctcgagtgcggccgcgaattcTTACGCGGCATTGCCCCAGG	R primer to amplify P131_09535
13	06853-F	ccaattcagtcgactggatccATGAAGTACACCAGCGCCAT	F primer to amplify P131_06853
14	06853-R	ctcgagtgcggccgcgaattcCTACTCGTCATCCTCCTCGT	R primer to amplify P131_06853
15	03335-F	ccaattcagtcgactggatccATGCAGTTCTCTTTCGCCAC	F primer to amplify P131_03335
16	03335-R	ctcgagtgcggccgcgaattcTTAGTTGACTCTGGAGTCCG	R primer to amplify P131_03335
17	09450-F	ccaattcagtcgactggatccATGAGAGTCAGCACCGCCTT	F primer to amplify P131_09450
18	09450-R	ctcgagtgcggccgcgaattcTCAATCCACGCACATGATCT	R primer to amplify P131_09450
19	09587-F	ccaattcagtcgactggatccATGGTTCGCGTTTCCACTTT	F primer to amplify P131_09587
20	09587-R	ctcgagtgcggccgcgaattcTTAGAAACCCTGCTTCTTG	R primer to amplify P131_09587
21	06247-F	ccaattcagtcgactggatccATGAAGACTTCAATTGCCGC	F primer to amplify P131_06247
22	06247-R	ctcgagtgcggccgcgaattcTCATGAACAAGGATTGTTA	R primer to amplify P131_06247
23	02098-F	ccaattcagtcgactggatccATGCAGTTCTTCAGCGTCGC	F primer to amplify P131_02098
24	02098-R	ctcgagtgcggccgcgaattcTTACTCCTCATCCTCATCC	R primer to amplify P131_02098
25	11979-F	ccaattcagtcgactggatccATGCAGTTCTCAACCATCAT	F primer to amplify P131_11979
26	11979-R	ctcgagtgcggccgcgaattcCTATTGGCGCTGAGCCTGA	R primer to amplify P131_11979
27	06308-F	ccaattcagtcgactggatccATGCACGTCAAGACCACCTG	F primer to amplify P131_06308
28	06308-R	ctcgagtgcggccgcgaattcTTACTCCTTTCCCTCGTTGC	R primer to amplify P131_06308
29	05316-F	ccaattcagtcgactggatccATGAAGACTTCCACCATT	F primer to amplify P131_05316
30	05316-R	ctcgagtgcggccgcgaattcTTATGTGTGGCCGTAGGCT	R primer to amplify P131_05316
31	07132-F	ccaattcagtcgactggatccATGCTTGCCAACAACATCCT	F primer to amplify P131_07132
32	07132-R	ctcgagtgcggccgcgaattcTTATGGTTTCGCACTGCAC	R primer to amplify P131_07132
33	08408-F	ccaattcagtcgactggatccATGCAGTTCAGCAGCGTCTT	F primer to amplify P131_08408
34	08408-R	ctcgagtgcggccgcgaattcTTAAGCGTCGGCCAGGTTGC	R primer to amplify P131_08408
35	07667-F	ccaattcagtcgactggatccATGAATTTCCTCATCCTCC	F primer to amplify P131_07667
36	07667-R	ctcgagtgcggccgcgaattcTCAGCTAGCTGACCTTGCC	R primer to amplify P131_07667
37	08608-F	ccaattcagtcgactggatccATGAAGGCTTACGGTATCT	F primer to amplify P131_08608
38	08608-R	ctcgagtgcggccgcgaattcTTAAAAGCAGGAGGCATTG	R primer to amplify P131_08608
39	08852-F	ccaattcagtcgactggatccATGTACGCCTTCAACTTCT	F primer to amplify P131_08852
40	08852-R	ctcgagtgcggccgcgaattcTTAGGCCTTTGGAGGGCAGT	R primer to amplify P131_08852
41	04799-F	ccaattcagtcgactggatccATGCAACTCATCAACATCCT	F primer to amplify P131_04799
42	04799-R	ctcgagtgcggccgcgaattcTCAATGCAACAATGCTTGAT	R primer to amplify P131_04799
43	10540-F	ccaattcagtcgactggatccATGCAGCTTCAGTCAATCA	F primer to amplify P131_10540
44	10540-R	ctcgagtgcggccgcgaattcTCAAAATTTAGGATCGTGGC	R primer to amplify P131_10540
45	02490-F	ccaattcagtcgactggatccATGAAGTTCTCCATCGCCTC	F primer to amplify P131_02490
46	02490-R	ctcgagtgcggccgcgaattcTTAGTCTTGCTCGGCGGCGA	R primer to amplify P131_02490
47	10190-F	ccaattcagtcgactggatccATGCAGTTTCTCAGCACCA	F primer to amplify P131_10190

48	10190-R	ctcgagtgcggccgcgaattcTTACCAGTCCCAGCACTGG	R primer to amplify P131_10190
49	03396-F	ccaattcagtcgactggatccATGAAGTTCTCCACCACTGC	F primer to amplify P131_03396
50	03396-R	ctcgagtgcggccgcgaattcTCATCTTATAGATCTTTGTC	R primer to amplify P131_03396
51	04934-F	ccaattcagtcgactggatccATGAGAAATATCTTCAGCG	F primer to amplify P131_04934
52	04934-R	ctcgagtgcggccgcgaattcTTATTTGAGGTTCTCGGCAG	R primer to amplify P131_04934
53	10366-F	ccaattcagtcgactggatccATGACGGCTCTGCTCATCT	F primer to amplify P131_10366
54	10366-R	ctcgagtgcggccgcgaattcTCAAGGGTGAACTACCGCCA	R primer to amplify P131_10366
55	00603-F	ccaattcagtcgactggatccATGAAGTTCACCGCTCCCC	F primer to amplify P131_00603
56	00603-R	ctcgagtgcggccgcgaattcTTAACACGCCTTGTCGTCTTC	R primer to amplify P131_00603
57	12512-F	ccaattcagtcgactggatccATGATGCAAGTTCTCAAGT	F primer to amplify P131_12512
58	12512-R	ctcgagtgcggccgcgaattcCTAATTCTTTTCATGCCTA	R primer to amplify P131_12512
59	05618-F	ccaattcagtcgactggatccATGCAATTCTCAGTCCTTC	F primer to amplify P131_05618
60	05618-R	ctcgagtgcggccgcgaattcCTAGGTGCCTTTTTCAACAG	R primer to amplify P131_05618
61	09418-F	ccaattcagtcgactggatccATGGTTTCGCTCAGGTTCA	F primer to amplify P131_09418
62	09418-R	ctcgagtgcggccgcgaattcTTATGGCTTGGACTCGCAC	R primer to amplify P131_09418
63	04965-F	ccaattcagtcgactggatccATGTTCTCCAAAACATTGC	F primer to amplify P131_04965
64	04965-R	ctcgagtgcggccgcgaattcTTACTCGGCGTAGAACCAGT	R primer to amplify P131_04965
65	03746-F	ccaattcagtcgactggatccATGACAGTCCTCAAGCTTG	F primer to amplify P131_03746
66	03746-R	ctcgagtgcggccgcgaattcTCACAACAGCATGCCACCC	R primer to amplify P131_03746
67	08988-F	ccaattcagtcgactggatccATGGTTCGCCTGTCCACCC	F primer to amplify P131_08988
68	08988-R	ctcgagtgcggccgcgaattcTCAGGCAGTGCAGGAAAAG	R primer to amplify P131_08988
69	01991-F	ccaattcagtcgactggatccATGTTGAGCAATTTCGCAA	F primer to amplify P131_01991
70	01991-R	ctcgagtgcggccgcgaattcTCACAGAGCAAAGGCACCC	R primer to amplify P131_01991
71	05726-F	ccaattcagtcgactggatccATGTTTGAAAATTATCTAG	F primer to amplify P131_05726
72	05726-R	ctcgagtgcggccgcgaattcTCAACTCGGCTGTGACGGAT	R primer to amplify P131_05726
73	04657-F	ccaattcagtcgactggatccATGAAGTCCCTCTTCCTCAC	F primer to amplify P131_04657
74	04657-R	ctcgagtgcggccgcgaattcTTAAATGTATTGCCAAGCCG	R primer to amplify P131_04657
75	10498-F	ccaattcagtcgactggatccATGCAGCTCGTCACCCTT	F primer to amplify P131_10498
76	10498-R	ctcgagtgcggccgcgaattcTCAAGCATCGCTGAGGAGGA	R primer to amplify P131_10498
77	05053-F	ccaattcagtcgactggatccATGATCGGTGTCAAGGCAG	F primer to amplify P131_05053
78	05053-R	ctcgagtgcggccgcgaattcCTAGTCCAGGTTGCCAGGC	R primer to amplify P131_05053
79	11027-F	ccaattcagtcgactggatccATGGCTTCGAAATATTTA	F primer to amplify P131_11027
80	11027-R	ctcgagtgcggccgcgaattcTTAGTCGTCATTTCTACCAG	R primer to amplify P131_11027
81	11327-F	ccaattcagtcgactggatccATGGCGCTGTTCGCAGGAG	F primer to amplify P131_11327
82	11327-R	ctcgagtgcggccgcgaattcTCACAAGTCGCAGAAGAAG	R primer to amplify P131_11327
83	06526-F	ccaattcagtcgactggatccATGAAGTTGCTCAACATCG	F primer to amplify P131_06526
84	06526-R	ctcgagtgcggccgcgaattcTTACAACAGGACAAGACCG	R primer to amplify P131_06526
85	04616-F	ccaattcagtcgactggatccATGTCGCAGGTCTTGACGC	F primer to amplify P131_04616
86	04616-R	ctcgagtgcggccgcgaattcTCACCGCATTAATAGTAATA	R primer to amplify P131_04616
87	05913-F	ccaattcagtcgactggatccATGGCTGCAACAAGGCGTTC	F primer to amplify P131_05913
88	05913-R	ctcgagtgcggccgcgaattcTCAGACCGCCCTCGGCTTTG	R primer to amplify P131_05913
89	10596-F	ccaattcagtcgactggatccATGGGCAAAGACACCAAGG	F primer to amplify P131_10596
90	10596-R	ctcgagtgcggccgcgaattcTTACTCGTCCTGCATCAGGG	R primer to amplify P131_10596
91	01240-F	ccaattcagtcgactggatccATGAAGTTCTTGGCTCTCC	F primer to amplify P131_01240
92	01240-R	ctcgagtgcggccgcgaattcTCAAATGTAGTTGCCCTT	R primer to amplify P131_01240
93	10222-F	ccaattcagtcgactggatccATGTTCGCCAAGGTTACCA	F primer to amplify P131_10222
94	10222-R	ctcgagtgcggccgcgaattcTTACTCCTCGTCGTCCTCA	R primer to amplify P131_10222
95	02819-F	ccaattcagtcgactggatccATGCACTTCTCCACCACCG	F primer to amplify P131_02819
96	02819-R	ctcgagtgcggccgcgaattcCTAAGGAGCGCACAGCGTCA	R primer to amplify P131_02819

97	08949-F	ccaattcagtcgactggatccATGGCACCCCTCACGCACC	F primer to amplify P131_08949
98	08949-R	ctcgagtgcggccgcgaattcTCAGCACCTCCTCCACTTG	R primer to amplify P131_08949
99	01166-F	ccaattcagtcgactggatccATGCGATTCTCCAGCCTCAC	F primer to amplify P131_01166
100	01166-R	ctcgagtgcggccgcgaattcTCAGGTGTGTGGCCATGGG	R primer to amplify P131_01166
101	01375-F	ccaattcagtcgactggatccATGCAATTCCCATCCGCTC	F primer to amplify P131_01375
102	01375-R	ctcgagtgcggccgcgaattcCTACATACAATCAGAAGCG	R primer to amplify P131_01375
103	01161-F	ccaattcagtcgactggatccATGCGCTTCAACTCGATCAC	F primer to amplify P131_01161
104	01161-R	ctcgagtgcggccgcgaattcTTACAGAAGAGCAGCAAGAC	R primer to amplify P131_01161
105	04833-F	ccaattcagtcgactggatccATGCATTCCACCACAAGTC	F primer to amplify P131_04833
106	04833-R	ctcgagtgcggccgcgaattcTTACAAAGCAGCCACAGCCC	R primer to amplify P131_04833
107	10254-F	ccaattcagtcgactggatccATGTACCTCATCAACCTGA	F primer to amplify P131_10254
108	10254-R	ctcgagtgcggccgcgaattcCTACTTGCATGACAGGCTG	R primer to amplify P131_10254
109	08851-F	ccaattcagtcgactggatccATGCCGTCCACGCTCACCC	F primer to amplify P131_08851
110	08851-R	ctcgagtgcggccgcgaattcTCATGCAATCGCAGTCATC	R primer to amplify P131_08851
111	02351-F	ccaattcagtcgactggatccATGAAGCTACAACAGACCGC	F primer to amplify P131_02351
112	02351-R	ctcgagtgcggccgcgaattcCTAAAGCAAGGCCGCGAG	R primer to amplify P131_02351
113	01889-F	ccaattcagtcgactggatccATGAAGTACTCAAAAGTCTC	F primer to amplify P131_01889
114	01889-R	ctcgagtgcggccgcgaattcCTATTCGGCACTCCTGCTCTC	R primer to amplify P131_01889
115	08602-F	ccaattcagtcgactggatccATGCTCATCTCGCGCCTC	F primer to amplify P131_08602
116	08602-R	ctcgagtgcggccgcgaattcTCAACCGGCAGAGCAGGTG	R primer to amplify P131_08602
117	04259-F	ccaattcagtcgactggatccATGCGCACCTTTTCTCTC	F primer to amplify P131_04259
118	04259-R	ctcgagtgcggccgcgaattcTCAGTTTCCGTCCGACTCG	R primer to amplify P131_04259
119	08153-F	ccaattcagtcgactggatccATGAAGTGCATCGCCAGC	F primer to amplify P131_08153
120	08153-R	ctcgagtgcggccgcgaattcTCAGCAGGTAAAGACGGGA	R primer to amplify P131_08153
121	02766-F	ccaattcagtcgactggatccATGCTCCCCTCGTGCATC	F primer to amplify P131_02766
122	02766-R	ctcgagtgcggccgcgaattcTCACAACACCCTCGTGCAAG	R primer to amplify P131_02766
123	02892-F	ccaattcagtcgactggatccATGTTACCCACCCTCCTG	F primer to amplify P131_02892
124	02892-R	ctcgagtgcggccgcgaattcTCAATGCGGTGCGTCGTT	R primer to amplify P131_02892
125	02630-F	ccaattcagtcgactggatccATGCAATTCAAATCCCTC	F primer to amplify P131_02630
126	02630-R	ctcgagtgcggccgcgaattcTCAAATGCCTATGCAATCC	R primer to amplify P131_02630
127	10151-F	ccaattcagtcgactggatccATGAGGGTCTCTCAGATCA	F primer to amplify P131_10151
128	10151-R	ctcgagtgcggccgcgaattcTTAGAGGATCAGGACCGCGC	R primer to amplify P131_10151
129	03327-F	ccaattcagtcgactggatccATGAAGTTTCAAATTCTC	F primer to amplify P131_03327
130	03327-R	ctcgagtgcggccgcgaattcTCATTGTTTGCCCTTCAAG	R primer to amplify P131_03327
131	09986-F	ccaattcagtcgactggatccATGGTCTCCTTCACCCAC	F primer to amplify P131_09986
132	09986-R	ctcgagtgcggccgcgaattcCTAGTTGCCGCTCTGGGGCT	R primer to amplify P131_09986
133	05921-F	ccaattcagtcgactggatccATGAAGTTCACCTCCACC	F primer to amplify P131_05921
134	05921-R	ctcgagtgcggccgcgaattcTTAGGCGCAGAGGGTAGCCT	R primer to amplify P131_05921
135	07649-F	ccaattcagtcgactggatccATGAAGGCCACACTCGTCAC	F primer to amplify P131_07649
136	07649-R	ctcgagtgcggccgcgaattcCTAGGCAAACCTCTTGCACG	R primer to amplify P131_07649
137	05538-F	ccaattcagtcgactggatccATGAAGTACGGATCTATCC	F primer to amplify P131_05538
138	05538-R	ctcgagtgcggccgcgaattcTCAGCACCATTCGCTGTGTG	R primer to amplify P131_05538

139	NbACT-RT-F	ATGGCAGACGGTGAGGATATTC	F primer for RT-PCR of *actin* gene of *N.b*
140	NbACT-RT-R	GCCTTTGCAATCCACATCTGTTG	R primer for RT-PCR of *actin* gene of *N.b*
141	NLP1-RT-F	GACGTCATCAACCACGATGC	F primer for RT-PCR of MoNLP1
142	NLP1-RT-R	GATGACGTTCTCCCAGTCGT	R primer for RT-PCR of MoNLP1
143	CEP1-RT-F	ATGAAGTTCACCGCTCCCCT	F primer for RT-PCR of MoCEP1
144	CEP1-RT-R	ACACGCCTTGTCGTCTTCCA	R primer for RT-PCR of MoCEP1
145	CEP2-RT-F	ATGGCAACTTCCTATGCCCC	F primer for RT-PCR of MoCEP2

146	CEP2-RT-R	TGACCTCGACGTTCCAGTTG	R primer for RT-PCR of MoCEP2
147	CEP3-RT-F	ATGCAGTTCTTCAGCGTCGC	F primer for RT-PCR of MoCEP3
148	CEP3-RT-R	CTCCTCATCCTCATCCTCCT	R primer for RT-PCR of MoCEP3
149	CEP4-RT-F	TCGTCAGACAACTGCACCAA	F primer for RT-PCR of MoCEP4
150	CEP4-RT-R	CTTGCGCCCTCCAAATTTCC	R primer for RT-PCR of MoCEP4
151	CEP5-RT-F	ATGAAGGCTTACGGTATCTT	F primer for RT-PCR of MoCEP5
152	CEP5-RT-R	AAAGCAGGAGGCATTGAGGT	R primer for RT-PCR of MoCEP5
153	CEP6-RT-F	CTGTGCACCAACTCCAACTG	F primer for RT-PCR of MoCEP6
154	CEP6-RT-R	TAGCTGGTGACCCTGTCGTT	R primer for RT-PCR of MoCEP6
155	CEP7-RT-F	CGCTGACGGAAATGCAACAC	F primer for RT-PCR of MoCEP7
156	CEP7-RT-R	AGTGGTGACCCAGGAACTTG	R primer for RT-PCR of MoCEP7
157	CEP8-RT-F	AGAATGGGCGTCATCTACCC	F primer for RT-PCR of MoCEP8
158	CEP8-RT-R	CACTCGGTGTTTTGCCTGTC	R primer for RT-PCR of MoCEP8
159	GAS1-RT-F	TCTCAAGAGCTTGATCGCCG	F primer for RT-PCR of MoGAS1
160	GAS1-RT-R	CACCGTTGACCTGGTGAAGA	R primer for RT-PCR of MoGAS1
161	BAS2-RT-F	ATGGTTCGCGTTTCCACTT	F primer for RT-PCR of BAS2
162	BAS2-RT-R	GAAACCCTGCTTCTTGACCT	R primer for RT-PCR of BAS2
163	10540-RT-R	ATGCAGCTTCAGTCAATCA	F primer for RT-PCR of P131_10540
164	10540-RT-R	TCAAAATTTAGGATCGTGGC	R primer for RT-PCR of P131_10540

165	CEP1-qPCR-F	ACCGCTCCCCTCTTTCTAGG	F primer for qPCR of MoCEP1
166	CEP1-qPCR-R	AGTTTTTCCTTTTTCGCGGGT	R primer for qPCR of MoCEP1
167	CEP2-qPCR-F	ATGCCAACTACGACCCCAAG	F primer for qPCR of MoCEP2
168	CEP2-qPCR-R	CCCGAGTTGCACTGGTACAT	R primer for qPCR of MoCEP2
169	CEP3-qPCR-F	TCCCTAACGGAAAGACGTGC	F primer for qPCR of MoCEP3
170	CEP3-qPCR-R	CATCCTCACGGGCTTGAAGT	R primer for qPCR of MoCEP3
171	CEP4-qPCR-F	TTCAAAGTGCCCCTCGAGAC	F primer for qPCR of MoCEP4
172	CEP4-qPCR-R	GCCTGTTGCATCCAGGAGTA	R primer for qPCR of MoCEP4
173	CEP5-qPCR-F	TGAAGCTCCTCCGCAGAAAG	F primer for qPCR of MoCEP5
174	CEP5-qPCR-R	GCTATCAGTTGCATCGCACG	R primer for qPCR of MoCEP5
175	CEP6-qPCR-F	TCTACCTGTGCACCAACTCC	F primer for qPCR of MoCEP6
176	CEP6-qPCR-R	TCCGGCGTTGAAGTAGAAGC	R primer for qPCR of MoCEP6
177	CEP7-qPCR-F	GGCTCATGCTCAACGACAAC	F primer for qPCR of MoCEP7
178	CEP7-qPCR-R	GTGTAAGGCACACTGGCAAC	R primer for qPCR of MoCEP7
179	CEP8-qPCR-F	ACAGGTCTTGCGACAACCAA	F primer for qPCR of MoCEP8
180	CEP8-qPCR-R	GAACTCTGCGTTGGAGCTGA	R primer for qPCR of MoCEP8
181	GAS1-qPCR-F	AGCTCAACGACGATGCTTCT	F primer for qPCR of MoGAS1
182	GAS1-qPCR-R	ACGAGAGGGAAGTCGGTCAT	R primer for qPCR of MoGAS1
183	BAS2-qPCR-F	ACCGATTGCCAGTCGAGATG	F primer for qPCR of BAS2
184	BAS2-qPCR-R	TAACGGTCTGGGCCTTGTTG	R primer for qPCR of BAS2

185	FLCEP1-S-F	tttaattaagaattcATGAAGTTCACCGCTCCCCT	F primer of full length of MoCEP1 for yeast secretion
186	FLCEP1-S-R	agggagaacctcgagACACGCCTTGTCGTCTTCCA	R primer of full length of MoCEP1 for yeast secretion
187	FLCEP2-S-F	tttaattaagaattcATGAAGTCCCTCTTCCTCAC	F primer of full length of MoCEP2 for yeast secretion
188	FLCEP2-S-R	agggagaacctcgagAATGTATTGCCAAGCCGAAG	R primer of full length of MoCEP2 for yeast secretion
189	FLCEP3-S-F	tttaattaagaattcATGCAGTTCTTCAGCGTCGC	F primer of full length of MoCEP3 for yeast secretion

190	FLCEP3-S-R	agggagaacctcgagCTCCTCATCCTCATCCTCCT	R primer of full length of MoCEP3 for yeast secretion
191	FLCEP5-S-F	tttaattaagaattcATGAAGGCTTACGGTATCTT	F primer of full length of MoCEP5 for yeast secretion
192	FLCEP5-S-R	agggagaacctcgagAAAGCAGGAGGCATTGAGGTC	R primer of full length of MoCEP5 for yeast secretion
193	FLCEP7-S-F	tttaattaagaattcATGTTCTCCAAAACATTGCT	F primer of full length of MoCEP7 for yeast secretion
194	FLCEP7-S-R	agggagaacctcgagCTCGGCGTAGAACCAGTAAA	R primer of full length of MoCEP7 for yeast secretion
195	NSCEP1-S-F	tttaattaagaattcATG GACCCCAACAGCCCTCACGA	F primer of MoCEP1 with no signal peptide for yeast secretion
196	NSCEP2-S-F	tttaattaagaattcATGGAAAAGTGCGGCGATGCCAAC	F primer of MoCEP2 with no signal peptide for yeast secretion
197	NSCEP3-S-F	tttaattaagaattcATGAAGGCGGTACGTGGAGCTAAT	F primer of MoCEP3 with no signal peptide for yeast secretion
198	NSCEP5-S-F	tttaattaagaattcATGCAAGGGGCCACAAACGAGGT	F primer of MoCEP5 with no signal peptide for yeast secretion
199	NSCEP7-S-F	tttaattaagaattcATGTCGCCTTGCAAGCCCAGGGC	F primer of MoCEP7 with no signal peptide for yeast secretion
200	SPCEP1-S-R	agggagaacctcgagAGGGCTGTTGGGGTCGCTCA	R primer of signal peptide of MoCEP7 for yeast secretion
201	SPCEP2-S-R	agggagaacctcgagTGCGAGAGCTGATGCACT	R primer of signal peptide of MoCEP7 for yeast secretion
202	SPCEP3-S-R	agggagaacctcgagGGCAGACACGGTGGCGGCCA	R primer of signal peptide of MoCEP7 for yeast secretion
203	SPCEP5-S-R	agggagaacctcgagAGCGGTTACGCCCACAGCAA	R primer of signal peptide of MoCEP7 for yeast secretion
204	SPCEP7-S-R	agggagaacctcgagGGCGGAAACAGAAGCGACGA	R primer of signal peptide of MoCEP7 for yeast secretion

205	CEP1-GTN-F	caccaaaccgccaaaggatccATGAAGTTCACCGCTCCCCT	F primer of MoCEP1 for secretion
206	CEP1-GTN-R	acctctagaactagtggatccACACGCCTTGTCGTCTTCCA	R primer of MoCEP1 for secretion
207	CEP2-GTN-F	caccaaaccgccaaaggatccATGAAGTCCCTCTTCCTCAC	F primer of MoCEP2 for secretion
208	CEP2-GTN-R	acctctagaactagtggatccAATGTATTGCCAAGCCGAAG	R primer of MoCEP2 for secretion
209	CEP3-GTN-F	caccaaaccgccaaaggatccATGCAGTTCTTCAGCGTCGC	F primer of MoCEP3 for secretion
210	CEP3-GTN-R	acctctagaactagtggatccCTCCTCATCCTCATCCTCCT	R primer of MoCEP3 or secretion
211	CEP5-GTN-F	caccaaaccgccaaaggatccATGAAGGCTTACGGTATCTT	F primer of MoCEP5 for secretion
212	CEP5-GTN-R	acctctagaactagtggatccAAAGCAGGAGGCATTGAGGT	R primer of MoCEP5 for secretion
213	CEP7-GTN-F	caccaaaccgccaaaggatccATGTTCTCCAAAACATTGCT	F primer of MoCEP7 for secretion
214	CEP7-GTN-R	acctctagaactagtggatccCTCGGCGTAGAACCAGTAAA	R primer of MoCEP7 for secretion

215	CEP1-LB-F	cgctctagaactagtggatccAGTGTCAGCATTGTCGGTGCC	F primer for making Δ-Mocep1 mutant
216	CEP1-LB-R	tcagttatcgaattcctgcagGCGCTAGTTTTCAGGCAGTCC	R primer for making Δ-Mocep1 mutant
217	CEP1-RB-F	cgggaaccagttatcaagcttGAATGGAATGTAGGGCGAACTC	F primer for making Δ-Mocep1 mutant
218	CEP1-RB-R	ggtaccgggccccccctcgagACAATCCGAATGCTGGGTGA	R primer for making Δ-Mocep1 mutant
219	CEP2-LB-F	cgctctagaactagtggatccCTCTGTAGGGCTGAGTTGAAGTTG	F primer for making Δ-Mocep2 mutant
220	CEP2-LB-R	tcagttatcgaattcctgcagGGTTACTTTGGCGGTGTGATG	R primer for making Δ-Mocep2 mutant
221	CEP2-RB-F	ccagttatcaagcttatcgatAGAGTTTGGCTGTAAGAGAAGGTGA	F primer for making Δ-Mocep2 mutant
222	CEP2-RB-R	ggtaccgggccccccctcgagGTTGACCGAACCTTGAATCAACC	R primer for making Δ-Mocep2 mutant
223	CEP3-LB-F	cgctctagaactagtggatccGCCATCAACACCATCTCTGGC	F primer for making Δ-Mocep3 mutant
224	CEP3-LB-R	tcaatatcagttatcgaattcGTAGACTGTTGTGGGTTGTGTTGG	R primer for making Δ-Mocep3 mutant
225	CEP3-RB-F	cgggaaccagttatcaagcttTTATGCTTGGGTTTCGGCAA	F primer for making Δ-Mocep3 mutant
226	CEP3-RB-R	ggtaccgggccccccctcgagAGATGATGACCGAGGTGAGTCG	R primer for making Δ-Mocep3 mutant
227	CEP5-LB-F	cgctctagaactagtggatccCCTAATCACGAACTGCCATGTTT	F primer for making Δ-Mocep5 mutant
228	CEP5-LB-R	tcaatatcagttatcgaattcGATTGTGTTGTGTGGATTGGATG	R primer for making Δ-Mocep5 mutant
229	CEP5-RB-F	cgggaaccagttatcaagcttTTGCTGGAGAGGTGGATTCTG	F primer for making Δ-Mocep5 mutant
230	CEP5-RB-R	ggtaccgggccccccctcgagGCTTGCTGGGTTTGGAATAAAC	R primer for making Δ-Mocep5 mutant
231	CEP7-LB-F	cgctctagaactagtggatccTACTGTCCGTCACCAAGCATTC	F primer for making Δ-Mocep7 mutant
232	CEP7-LB-R	tcaatatcagttatcgaattcTGACTTTGGGTGTGTGGATGG	R primer for making Δ-Mocep7 mutant
233	CEP7-RB-F	aagcttatcgataccgtcgacCGCCATCACTTTCTTCGTCTCT	F primer for making Δ-Mocep7 mutant
234	CEP7-RB-R	agggaacaaaagctgggtaccTTGAGTTCAGCAGCGAAACAAAT	R primer for making Δ-Mocep7 mutant

235	CEP1-Probe-F	AGTGTCAGCATTGTCGGTGCC	F primer for probe of MoCEP1
236	CEP1-Probe-R	GCGCTAGTTTTCAGGCAGTCC	F primer for probe of MoCEP1
237	CEP2-Probe-F	CTCTGTAGGGCTGAGTTGAAGTTG	F primer for probe of MoCEP2
238	CEP2-Probe-R	GGTTACTTTGGCGGTGTGATG	F primer for probe of MoCEP2
239	CEP3-Probe-F	TTATGCTTGGGTTTCGGCAA	F primer for probe of MoCEP3
240	CEP3-Probe-R	AGATGATGACCGAGGTGAGTCG	F primer for probe of MoCEP3
241	CEP5-Probe-F	CCTAATCACGAACTGCCATGTTT	F primer for probe of MoCEP5
242	CEP5-Probe-R	GATTGTGTTGTGTGGATTGGATG	F primer for probe of MoCEP5
243	CEP7-Probe-F	CGCCATCACTTTCTTCGTCTCT	F primer for probe of MoCEP7
244	CEP7-Probe-R	TTGAGTTCAGCAGCGAAACAAAT	F primer for probe of MoCEP7
