# Supplementary figures and images for: Identification and Characterization of Novel Candidate Effector Proteins from Magnaporthe oryzae
Source: J Fungi (Basel). 2023 May 15;9(5):574. doi: 10.3390/jof9050574 (PMC10219149; doi:10.3390/jof9050574)

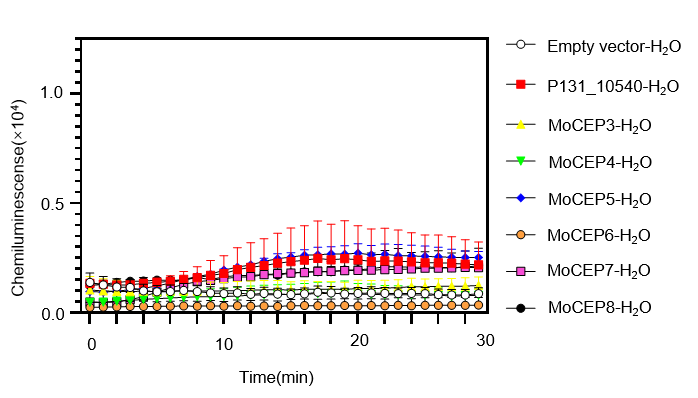

Supplement: Supplementary file 1 [file jof-09-00574-s001.zip › Figure S1.tif]

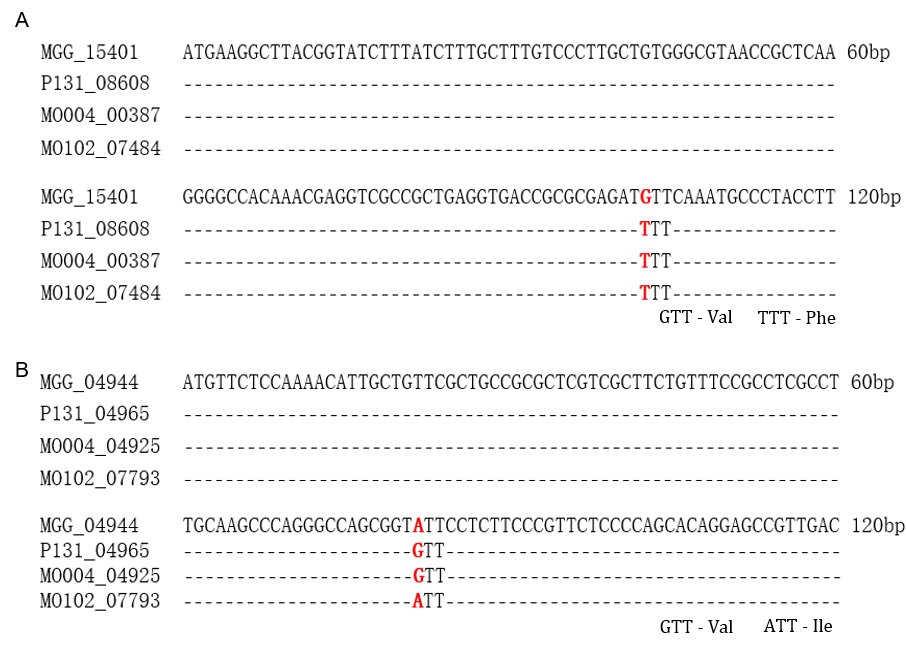

Supplement: Supplementary file 1 [file jof-09-00574-s001.zip › Figure S2.tif]

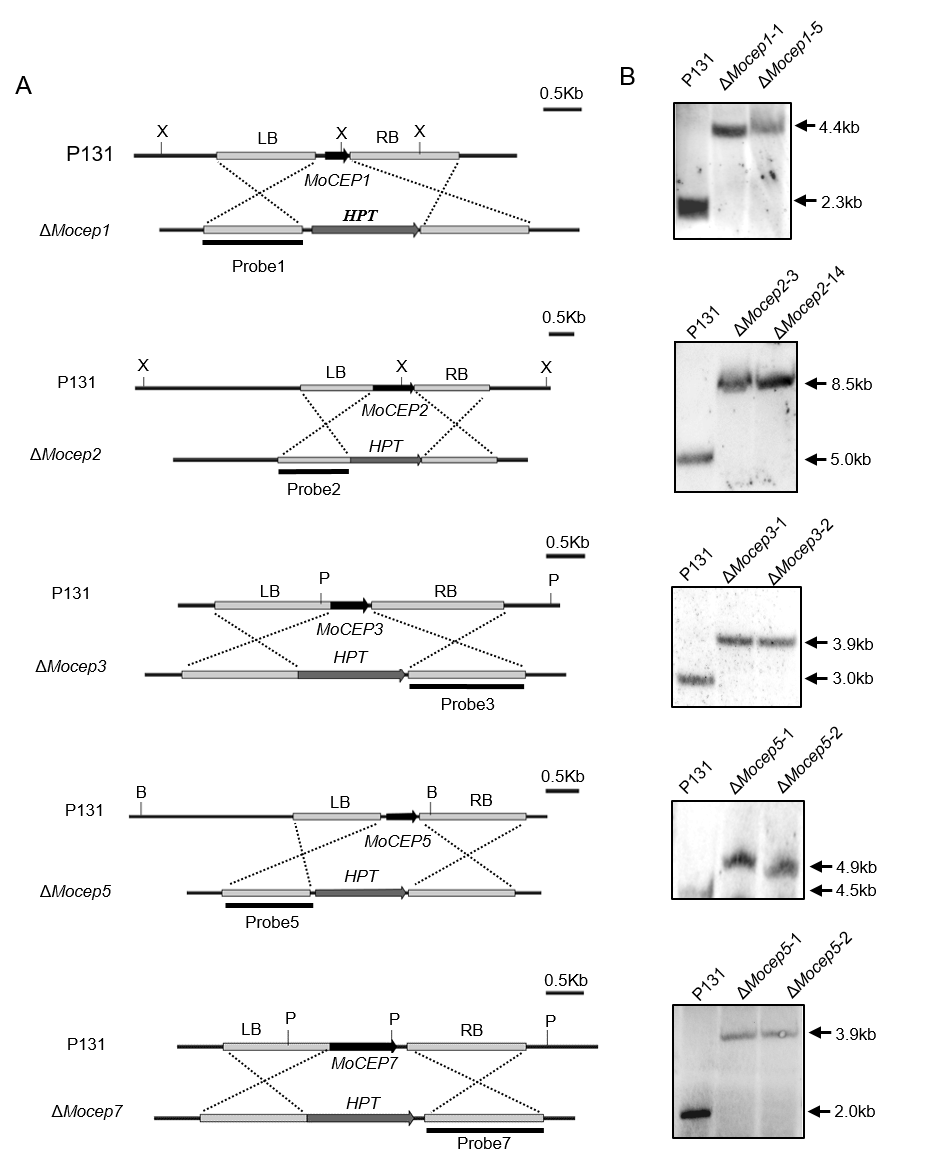

Supplement: Supplementary file 1 [file jof-09-00574-s001.zip › Figure S3.tif]

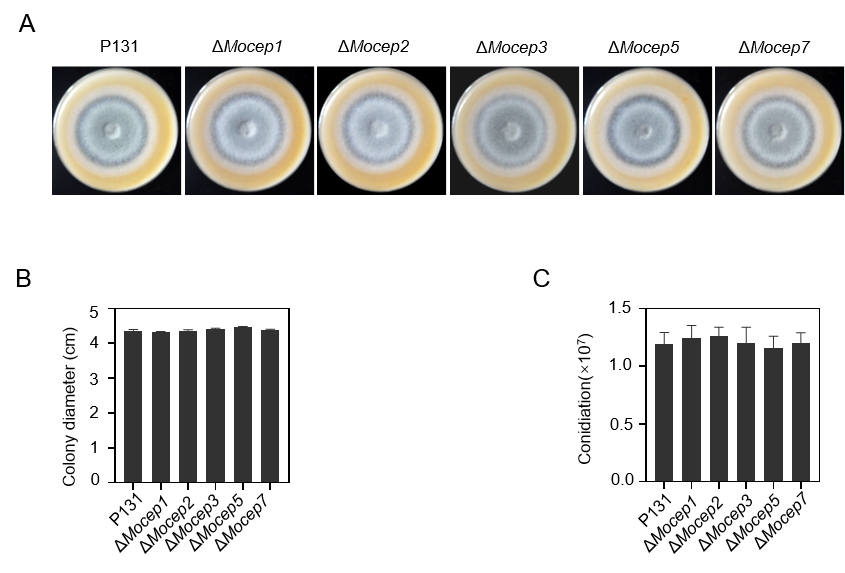

Supplement: Supplementary file 1 [file jof-09-00574-s001.zip › Figure S4.tif]
